# Cap-Gly Proteins at Microtubule Plus Ends: Is EB1 Detyrosination Involved?

**DOI:** 10.1371/journal.pone.0033490

**Published:** 2012-03-14

**Authors:** Anouk Bosson, Jean-Marc Soleilhac, Odile Valiron, Didier Job, Annie Andrieux, Marie-Jo Moutin

**Affiliations:** Institut National de la Santé et de la Recherche Médicale (INSERM) U836, Grenoble Institut des Neurosciences, Commissariat à l'Energie Atomique (CEA) Institut de Recherches en Technologies et Sciences pour le Vivant - Groupe Physiopathologie du Cytosquelette (iRTSV-GPC), Université Joseph Fourier, Grenoble, France; Université de Genève, Switzerland

## Abstract

Localization of CAP-Gly proteins such as CLIP170 at microtubule+ends results from their dual interaction with α-tubulin and EB1 through their C-terminal amino acids −EEY. Detyrosination (cleavage of the terminal tyrosine) of α-tubulin by tubulin-carboxypeptidase abolishes CLIP170 binding. Can detyrosination affect EB1 and thus regulate the presence of CLIP170 at microtubule+ends as well? We developed specific antibodies to discriminate tyrosinated vs detyrosinated forms of EB1 and detected only tyrosinated EB1 in fibroblasts, astrocytes, and total brain tissue. Over-expressed EB1 was not detyrosinated in cells and chimeric EB1 with the eight C-terminal amino acids of α-tubulin was only barely detyrosinated. Our results indicate that detyrosination regulates CLIPs interaction with α-tubulin, but not with EB1. They highlight the specificity of carboxypeptidase toward tubulin.

## Introduction

Microtubules are hollow tubes composed of α- and β-tubulin heterodimers, which are essential for cell morphogenesis, division, and motility and for intracellular motile events. They are intrinsically polar structures with a predominantly stable minus end and a highly dynamic plus end. Several proteins called plus-end tracking proteins (+TIPs) associate specifically with growing microtubule+ends where they regulate microtubule dynamic parameters and interactions with other cellular elements like organelles, cell cortex, or actin filaments [1], [2]. +TIPs can bind to each other and form dynamic complexes at microtubule ends. Among +TIPs, EB1 is a small homodimeric protein of 30 kDa considered to form the core of the microtubule+end complexes. EB1 interacts directly through a consensus polypeptide motif Ser-x-Ile-Pro (SxIP) with the majority of the other known +TIPs, recruiting them to microtubule+ends [2], [3]. However, the CAP-Gly-containing proteins (CLIP-170, CLIP-115, p150Glued), which are also targeted to microtubule+ends by EB1, bind differently to this core protein. The interaction between EB1 and the CLIPs occurs through the C-terminus part of EB1 which terminates in an acidic tail with the amino acids sequence −EEY [4], [5]. It has been shown *in vitro* that this interaction requires the terminal aromatic residue tyrosine of EB1, since a mutant form of EB1 lacking the tyrosine only weakly binds to CLIP170 [6] and an EB1 mutant in which the tyrosine was replaced by an alanine is unable to bind CLIP-170 [7].

Besides its localization at microtubule+ends via EB1, CLIP-170 was also shown to directly recognize and bind to microtubules+ends in mammals and in *S. Cerevisiae* [8], [9], [10]. This direct interaction involves the C-terminal region of α-tubulin via a small C-terminal −EEY motif which displays a striking homology with the C-terminus of EB1. Importantly the last tyrosine residue of α-tubulin has been shown to be crucial for CLIP 170 binding. Indeed, α-tubulin is subjected to a detyrosination-tyrosination cycle in which the terminal tyrosine is removed by a carboxypeptidase (TCP) and re-added by Tubulin Tyrosine Ligase (TTL). In TTL deficient cells, abnormally enriched in detyrosinated tubulin, CLIP 170 binding to microtubules+ends is heavily reduced [9]. Similarly, in a *S. Cerevisiae* mutant strain expressing a detyrosinated form of α-tubulin, the homologue of CLIP-170 (Bik1p) cannot properly track microtubule ends [8]. Thus, CLIP 170 interaction with both EB1 and α-tubulin involves their −EEY C-terminal motifs and particularly their last tyrosine residue. The presence of CLIPs at microtubule+ends, which depend on their interaction with α-tubulin and with EB1, is necessary for normal microtubules interactions with the cell cortex; these connections being crucial for spindle positioning during mitosis and for neuronal polarization [9], [11]. Defective interactions of CLIPs at microtubules+ends may thus at least partly explain tumor progression of numerous cancers involving cells which lack TTL enzyme [12]. They also give molecular explanation for deleterious neuronal disorganization of TTL-deficient mouse [13].

As detyrosination of α-tubulin is one way to regulate the presence of CLIP170 protein at microtubule+end, we wondered whether EB1 detyrosination could also control CLIP 170 targeting, i.e. if EB1 could be detyrosinated in cells. To determine the possible occurrence of detyrosinated EB1, we developed highly specific anti-peptide antibodies directed against the tyrosinated form and presumed detyrosinated form of EB1. We show that mouse fibroblasts, astrocytes, and brain do not contain detectable amounts of detyrosinated EB1, even when we used TTL-deficient cells or tissue. We show that changing the eight C-terminal residues of EB1 with those of α-tubulin leads only to a near to the ground detyrosination of EB1 chimera. These results strongly suggest that EB1 is never detyrosinated in normal tissues and indicate that EB1 interaction with CLIPs is not regulated by detyrosination. These results exclude an involvement of EB1 detyrosination to explain TTL-deficient cell and mouse phenotypes. Additionally, our results highlight the high specificity of tubulin carboxypeptidase toward tubulin.

## Results and Discussion

### Specific antibodies against tyrosinated EB1 and putative detyrosinated EB1

To have tools to study the possible occurrence of detyrosinated EB1 in cells and tissues, we developed polyclonal antibodies directed against antigenic peptides designed from the eight terminal amino acids of EB1 with and without the terminal tyrosine: PQEEQEEY (for anti-tyrosinated EB1 named anti-Tyr EB1) and GPQEEQEE (for anti-detyrosinated EB1 named anti-deTyr EB1). Sera from rabbits for anti-Tyr EB1 and from guinea pigs for anti-deTyr EB1 were tested for dilution and specificity by both Western blotting and immunocytochemistry.

**Figure 1:**
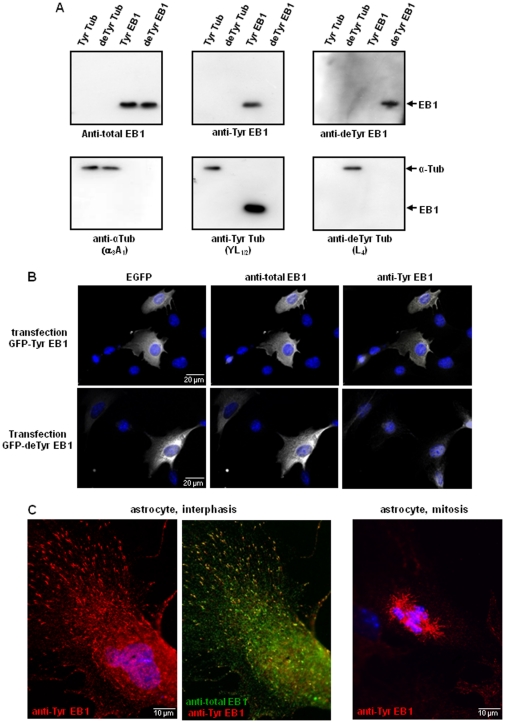
Analysis of developed anti-EB1 antibodies compared to the known anti-tubulin antibodies. (A) Western-blot analysis of the indicated proteins (15 ng) separated on 10% SDS-PAGE using a commercial anti-EB1 antibody (anti-total EB1, raised against amino-acids 107–268 of mouse EB1), the presently developed antibodies (anti-Tyr EB1 and anti-deTyr EB1), and tubulin antibodies. Detyrosinated EB1 was obtained from recombinant EB1 using carboxypeptidase A. Tyrosinated and detyrosinated tubulin were obtained from purified brain tubulin, using respectively TTL and carboxypeptidase A. Both anti-Tyr EB1 and anti-deTyr EB1 are highly specific. (B) Double immunostaining with anti-total EB1 antibody and anti-Tyr EB1 on fibroblasts after transfection of plasmids allowing expression of either tyrosinated or detyrosinated EB1 with EGFP at the N-terminus. The transfected cells were detected by EGFP signal. Anti-Tyr EB1 is highly specific of tyrosinated form of EB1. (C) Immunostaining of endogenous EB1 in astrocytes with anti-Tyr EB1 and anti-total EB1.

Figure 1A shows Western-blots obtained with anti-Tyr EB1, anti-deTyr EB1, and a commercial anti-EB1 (raised against amino-acids 107–268 of mouse EB1). Antibodies were tested on purified tyrosinated EB1 (over-expressed in *E. coli*) and detyrosinated EB1 (obtained from expressed tyrosinated EB1 using carboxypeptidase A). Whereas the commercial anti-EB1 (1∶2000) stained both forms of EB1, anti-Tyr EB1 (1∶15000) stained only tyrosinated EB1, and anti-deTyr EB1 (1∶200) stained only detyrosinated EB1. The commercial anti-EB1 was thus called anti-total EB1. To assay the specificity of the new EB1 antibodies, we also tested them on tyrosinated and detyrosinated forms of tubulin (obtained from mouse brain using tubulin tyrosine ligase and carboxypeptidase A). Although the EB1 and α-tubulin C-terminal sequences shared four amino acids out of eight, anti-Tyr EB1 and anti-deTyr EB1 did not react with tubulin. In the controls with tubulin antibodies (Fig. 1A lower part), we observed that YL_1/2_ [14] reacted not only with tyrosinated tubulin but also strongly with tyrosinated EB1 as expected [15]. Anti detyrosinated tubulin antibody [16], [17] is highly tubulin specific.

We then tested the developed antibodies in immunocytochemistry experiments. Fibroblasts were transfected with either tyrosinated EB1 or a mutant form of EB1 missing the last tyrosine (detyrosinated EB1), both fused to GFP at the N-terminus. Anti-Tyr EB1 (1∶1000) was shown to be highly specific: it strongly reacted with tyrosinated EB1 (GFP-Tyr EB1), but not with detyrosinated EB1 (GFP-deTyr EB1) (Figure 1B). Anti-deTyr EB1 was not reactive in immunocytochemistry experiments (data not shown).

Next, we analyzed the staining of endogenous EB1 within different mouse cell types. Anti-Tyr EB1 nicely stained all examined cells, including fibroblasts, astrocytes, and neurons. Figure 1C shows typical immunofluorescent labeling of endogenous EB1 from astrocytes obtained with anti-Tyr EB1 antibody (1∶1000). We observed selective accumulation of EB1 in comets at the microtubule plus ends during cell interphasis and decoration of mitotic spindle during cell mitosis. Staining of tyrosinated EB1 co-localized with staining with the commercial anti-EB1 antibody (anti-total EB1).

**Figure 2:**
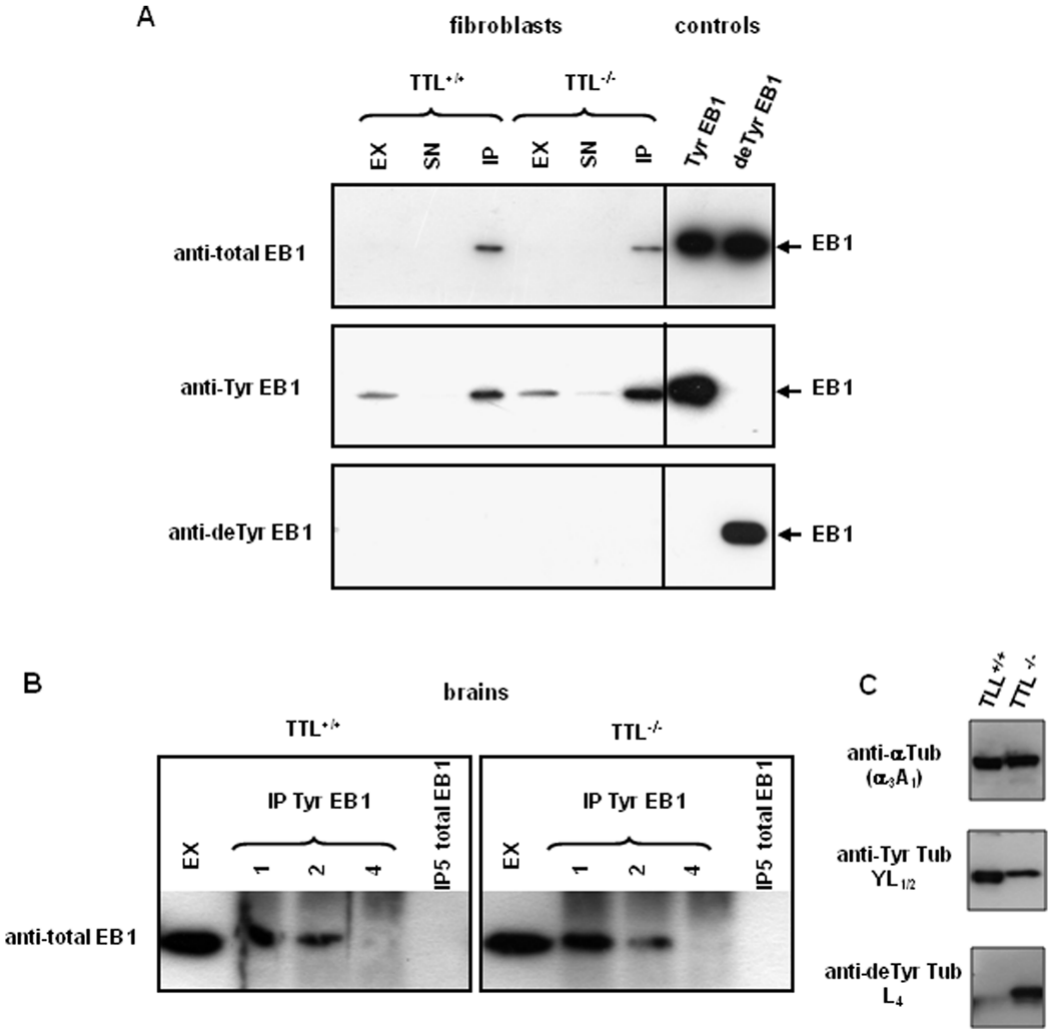
Study of endogenous EB1 C-termini in fibroblasts and brain from wild type and TTL-deficient mouse. Western-blot analysis of the indicated control proteins (see figure 1) or extracts. (A) Immunoprecipitation of endogenous EB1 from wild type (TTL^+/+^) or TTL-deficient MEFs using anti-total EB1 antibody, and analysis with anti-Tyr EB1 (1∶15000), anti-detyr EB1 (1∶200), and anti-total EB1 (1∶2000). EX: crude extract; SN: supernatant after immunoprecipitation; IP: immunoprecipitated fraction. No detyrosinated EB1 could be detected in the IP fractions. Note that anti-total EB1 antibody being less sensitive than anti-Tyr EB1, EB1 failed to be detected in crude extract (upper panel). (B) Immunodepletion of tyrosinated EB1 with anti-Tyr EB1 (IP 1 to 4) in brain extracts from wild type and TTL-knockout mice, followed by immunoprecipitation of the remaining EB1 with anti-total EB1 (IP5), and analysis with anti-total EB1 (1∶2000). No remaining EB1 could be detected after tyrosinated-EB1 immunodepletion. (C) Tyrosinated and detyrosinated tubulin pools in brain extracts from wild type and TTL-deficient mice were analyzed using anti-α tubulin (1∶10,000), anti-tyrosinated tubulin (YL_1/2_, 1∶20,000), and anti-detyrosinated tubulin (L_4_, 1∶20,000).

Altogether our results show that the antibodies we have developed against tyrosinated EB1 and putative detyrosinated EB1 are highly specific and are useful in Western-blot experiments. We then used them to examine the possible occurrence of the detyrosinated form of EB1 in mouse cells and tissues. We analyzed potential EB1 detyrosination in wild type fibroblasts and in brain, a tissue in which normally the majority of α tubulin is detyrosinated (see Figure 2C). As EB1 detyrosination could be mediated either by an unknown carboxypeptidase or by the TCP in unusual conditions where its natural substrate (tyrosinated tubulin) is largely decreased (see Figure 2C), we decided to also examine TTL-deficient fibroblast and brain.

### Endogenous EB1 is fully tyrosinated in wild type and TTL-deficient mouse fibroblasts and brain

We first prepared crude extracts of fibroblasts from wild type and TTL-deficient mice and concentrated their endogenous EB1 by immunoprecipitation using commercial anti-EB1 (which interacts equally with tyrosinated and detyrosinated EB1, as shown in Fig. 1A). Figure 2A clearly shows that EB1 from both cell types was entirely tyrosinated: whereas anti-Tyr EB1 signals were high in the immunoprecipitated fractions, no staining could be detected in these fractions with anti-deTyr EB1.

Next, we tested brain extracts from wild type and TTL-deficient mouse. After immunodepletion of Tyr EB1 using the antibody developed in the present work (IP1 to 4), we tested for the presence of the detyrosinated form of EB1 in the remaining extracts by immunoprecipitation with anti-total EB1 (IP5). Figure 2B shows that no other form of EB1 than tyrosinated EB1 could be detected in both wild type and TTL knock-out brain extracts.

Based on these data, we concluded that EB1 protein is fully tyrosinated in fibroblasts and brain from both wild type and TTL-deficient mice. EB1 C-terminus is not subjected to detyrosination even in TTL-deficient cells and in tissues in which the level of tyrosinated tubulin (the substrate of TCP) is decreased. To strengthen our results, we next decided to test if overexpressed EB1 could be modified in cells, and also if changing the eight C-terminal amino-acids PQEEQEEY of EB1 to α-tubulin amino-acids GEEEGEEY could lead to cellular detyrosination of chimeras.

### EB1 with the C-terminus of α-tubulin is barely detyrosinated

**Figure 3:**
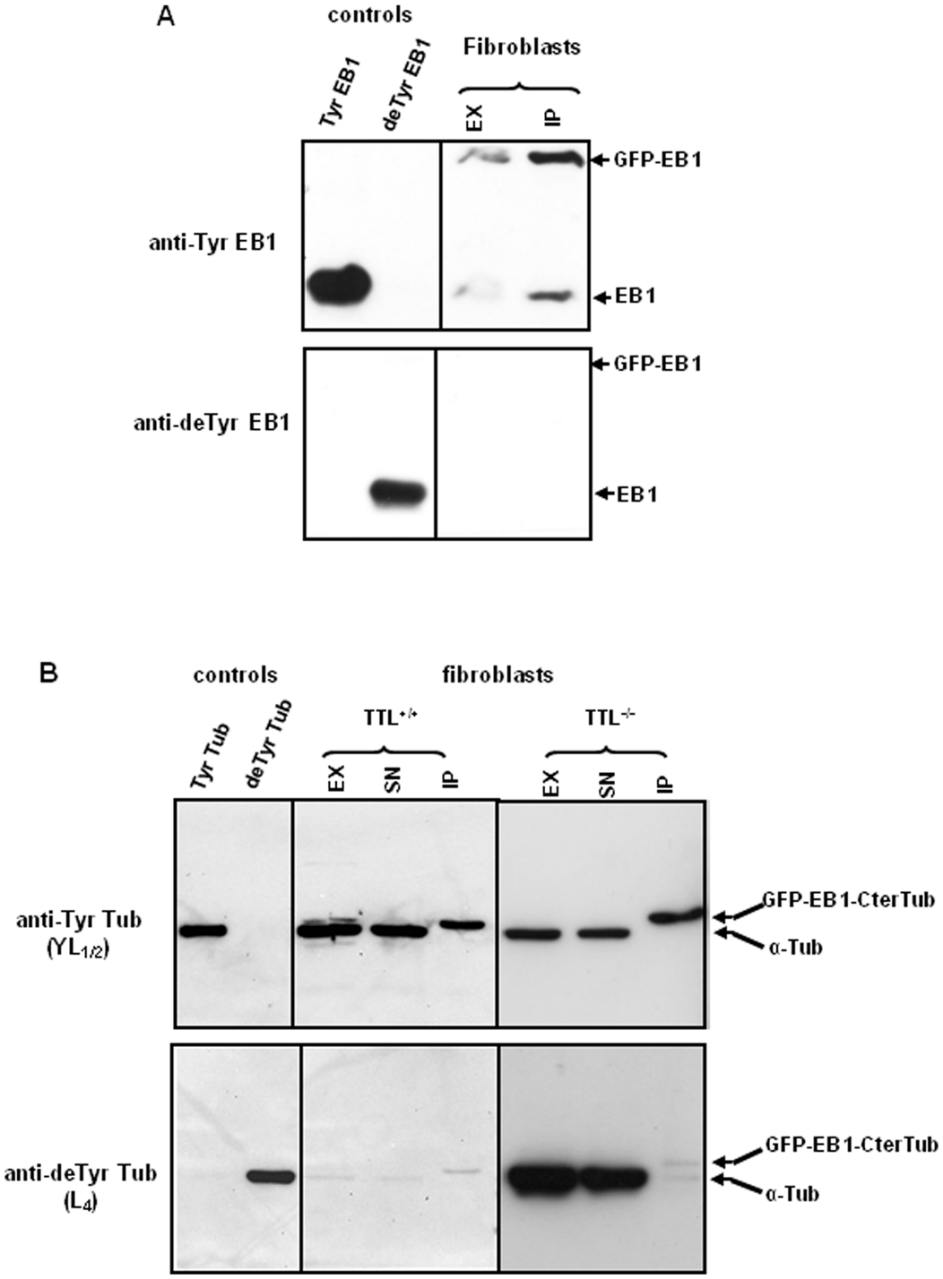
Analysis of C-termini of recombinant EB1 forms overexpressed in wild type and TTL-deficient fibroblasts. Western-blot analysis of the indicated control proteins (see figure 1) and of fractions of immunoprecipitation experiments carried out on cells transfected with cDNA encoding different EB1 forms. EX: crude extract; SN: supernatant after immunoprecipitation; IP: immunoprecipitated fraction. (A) transfection of fibroblasts (NIH3T3) with plasmids encoding tyrosinated EB1 fused with GFP at the N-terminus (GFP-EB1), followed by immunoprecipitation using anti-GFP antibody and analysis using anti-Tyr EB1 (1∶15000) and anti-deTyr EB1 (1∶200). No detyrosinated GFP-EB1 could be detected. (B) Transfection of fibroblasts with cDNA encoding GFP-EB1 ending with the C-terminus of α-tubulin GEEEGEEY (GFP-EB1-CterTub), followed by immunoprecipitation with anti-GFP antibody and analysis using anti-Tyr Tub (1∶20,000) and anti-deTyr Tub (1∶20,000). NIH3T3 were used as TTL^+/+^ cells and MEFs isolated from TTL null mice were used as TTL^−/−^. A very low quantity of detyrosinated protein ending with α-tubulin residues was detected (upper band in IP fractions of lower panel).

Fibroblasts (NIH3T3) were transfected with plasmids allowing expression of either GFP-EB1 (tyrosinated EB1 with GFP at the N-terminus) or GFP-EB1-CterTub (mutated EB1 with GFP at the N-terminus and ending with the amino-acids sequence of α-tubulin GEEEGEEY). After 48 hours of transfection, crude extracts of cells were prepared, and transfected proteins were immunoprecipitated using mouse anti-GFP. First, C-termini of GFP-EB1 proteins were analyzed by Western-blot using anti-Tyr EB1 and anti-deTyr EB1. Figure 3A presents a typical experiment that clearly shows that GFP-EB1 remained entirely tyrosinated in the fibroblasts. Note that endogenous EB1 co-immunoprecipitated with GFP-EB1. Second, C-termini of GFP-EB1-CterTub proteins were analyzed with antibodies against tubulin C-termini, anti-Tyr Tub (YL_1/2_), and anti-deTyr Tub (L_4_). In these experiments, endogenous tubulin was thus stained together with GFP-EB1-CterTub. Figure 3B presents the results obtained using both wild type (NIH3T3) and TTL-deficient fibroblasts. Anti-Tyr Tub antibody allows detection of Tyr Tub in supernatant and of GFP-EB1-Cter-Tub (having a slightly higher molecular weight) in immunoprecipitated (IP) fraction in both wild type and TTL-deficient cells. Anti deTyr Tub antibody allows detection of low levels of deTyr Tub in wild type cells and of large levels of deTyr Tub in TTl-deficient cells (see crude extracts and supernatants), as expected. We observed a very slight detyrosination of GFP-EB1-CterTub in these cells, comparable in TTL-deficient and wild type cells (see IP fractions, upper bands). The quantity of detyrosinated EB1-CterTub was estimated to represent less than 4% of overexpressed mutated EB1. The reactivity of the tubulin antibodies with GFP-EB1-CterTub was verified: after incubation of the immunoprecipitated extracts with carboxypeptidase A, the anti-Tyr Tub (YL_1/2_) signal completely disappeared, and staining was observed using anti-deTyr Tub (L_4_) (data not shown).

We concluded that EB1 is not a substrate for the tubulin-modifying enzyme TCP, and that a mutated EB1 that ends with the eight C-terminal residues of α-tubulin (EB1-CterTub) is a very poor substrate for this enzyme.

Altogether, our results establish that EB1 can be detected only on a tyrosinated form in the cells and tissues we tested. EB1 is fully tyrosinated in brain, a tissue in which tubulin is highly detyrosinated. Even in TTL null cells or tissue where the amount of normal TCP substrate (tyrosinated tubulin) is largely diminished, conditions favorable for TCP action on alternative substrates, we still only detected tyrosinated EB1. Hence, EB1 detyrosination seems unlikely involved in the defective recruitment of CAP-Gly proteins at microtubule plus ends in TTL null cells and thereby do no not contribute to the lethal phenotype observed in mice after TTL deletion.

To summarize, no enzyme, including TCP, seems capable of cleaving the C-terminal tyrosine of EB1 within cells. As a consequence, whereas detyrosination of the C-terminal part of α-tubulin by the TCP enzyme crucially regulates CLIP-170 localization at microtubules+ends, end-tracking of CLIP-170 via EB1 does not depend on EB1 detyrosination.

## Materials and Methods

### Antibodies

Polyclonal antibodies against tyrosinated EB1 (anti-Tyr EB1) were produced in rabbits by using peptide PQEEQEEY linked at its N-terminus to the Keyhole Limpet Hemocyanine (KLH) protein via a cystein. Polyclonal antibodies against detyrosinated EB1 (anti-deTyr EB1) were produced in guinea pigs by using KLH-CGPQEEQEE. Anti-total EB1 (mouse monoclonal IgG antibody against the C-terminal part of EB1, amino-acids 107–268) was from BD Biosciences. Antibodies specific to tyrosinated tubulin (mouse clone YL_1/2_; [14]) and detyrosinated tubulin (polyclonal antibody L_4_, developed in rabbits) were kind gifts of L. Lafanechère [17]. Antibody against α-tubulin was from mouse clone α3A1 [13]. Mouse anti-GFP was from Invitrogen. Anti-rabbit, anti-mouse, and anti-guinea pig antibodies tagged with either cyanine-5 or cyanine-3 were from Jackson Immuno Research, and anti-rabbit, anti-mouse and anti-guinea pig antibodies tagged with horse radish peroxidase (HRP) were from Macherey Nagel.

### Preparation of purified tubulin and EB1

Tyrosinated and detyrosinated tubulin were prepared according to [18]. In brief, tubulin was purified from bovine brains and incubated with either tubulin tyrosine ligase (kind gift of M. Steinmetz) to obtain tyrosinated tubulin (Tyr Tub) or carboxypeptidase A (Sigma) to obtain detyrosinated tubulin (deTyr Tub). EB1 (Tyr EB1) was obtained by overexpression of mouse EB1 fused to a 6-histidines cluster at its N-terminus in *E. coli* and purification on a nitrilotriacetate-Ni^2+^ adsorbent (Qiagen). Detyrosinated EB1 (deTyr EB1) was obtained by incubation of purified EB1 with carboxypeptidase A. Protein concentration was determined by the method of Bradford using bovine serum albumin as standard.

### Mouse Experiments

In compliance with the European Community Council Directive of November 24, 1986 (86/609/EEC), research involving animals has been authorized by the Direction Départementale de la Protection des Populations, Préfecture de l'Isère, France (permit n°38 07 11). Every effort was made to minimize the number of animals used and their suffering. This study has been approved by the local ethics committee of Grenoble Institut des Neurosciences. For tissue preparation or cell culture, mice were sacrificed and organs were quickly dissected.

### Cell culture and immunofluorescence

Hippocampal neurons and murine embryonic fibroblasts (MEFs) were prepared as previously described [13]. Astrocytes were obtained from cerebral cortices of 18-day mice fetuses. Cells were fixed by 10 min incubation with methanol at −20°C. They were then incubated with rabbit anti-Tyr EB1 (1∶2000), guinea-pig anti-deTyr EB1 (1/10 to 1/200), or mouse anti-total EB1 (1∶500, BD Transduction laboratories) for 1 h, followed by 30 min incubation with anti-rabbit, anti-guinea pig, or anti-mouse antibodies conjugated with either cyanine-3 or cyanine-5 fluorophores (1∶1,000; Molecular Probes). Nuclei were stained using Hoescht 33258 (1 µg/ml). Images were acquired using Axioskop 50 (Carl Zeiss MicroImaging) microscopes and Metamorph (Universal Imaging Corp., USA) software.

### Expression constructs and cell transfections

Vector pEGFP-C3 (BD bioscience Clontech) was used to construct plasmids allowing expression of the EB1 proteins fused to GFP at their N-terminus: GFP-Tyr EB1, GFP-deTyr EB1, and GFP-EB1-CterTub (with EB1 eight C-terminal amino-acids replaced by the residues of α-tubulin). In brief, cDNA of mouse EB1 (Tyr EB1) was obtained with mRNA prepared from mouse brain using Superscript One-Step RT-PCR kit (Invitrogen) and was cloned between SacI and SacII restriction sites of pEGFP-C3 to create pEGFP-Tyr EB1. This plasmid was modified using PCR (between HindIII and SacII restriction sites) to construct pEGFP-deTyr EB1 (missing the last codon coding for the tyrosine) and to create pEGFP-EB1-CterTub. All constructs were verified by restriction digestions and DNA sequencing analysis. Cells were then transfected using Nucleofector kits (Amaxa Biosystems).

### Immunoprecipitation and Immunoblotting

Fibroblasts and brain lysates were obtained by extraction in 50 mM Tris at pH 8.0, 150 mM NaCl, 0.05% deoxycholate, 1% Triton X-100, 10% glycerol, protease inhibitors (complete Mini EDTA free, Roche diagnostics), and DNase (0.1 µg/µL), followed by centrifugation (13,000 g 3 min). For immunoprecipitation, fibroblast lysates were incubated 90 min at 4°C in 50 mM Tris at pH 8.0, 150 mM NaCl, 0.04% deoxycholate, 0,8% Triton X-100, 8% glycerol, protease inhibitors, and DNase with either mouse anti-total EB1 or mouse anti-GFP antibodies pre-incubated overnight with Ultralink immobilized protein G beads (PIERCE). After centrifugation at 13,000 *g* for 2 min, the immunoprecipitates were washed three times with 50 mM Tris at pH 8.0, 150 mM NaCl, 0.05% Triton X-100, and protease inhibitors. For immunodepletion, brain lysates were incubated 90 min at 4°C in 50 mM Tris at pH 8.0, 150 mM NaCl, 0.03% deoxycholate, 0.6% Triton X-100, 6% glycerol, protease inhibitors, and DNase with anti-total EB1 antibody pre-incubated 60 min with protein G beads (PIERCE). After centrifugation at 13,000 *g* for 3 min, the supernatant was used for a novel immunoprecipitation with the same antibody in the same conditions, and this was repeated three times (IP1-4). The fifth immunoprecipitation (IP5) was then performed in the same experimental conditions with anti-Tyr EB1. Proteins were resolved on 10% SDS-PAGE followed by electrotransfer to Immobilon P sheets (Millipore). Primary antibodies (see Fig. 2 and Fig. 3) were incubated and the blots were then stained with secondary antibodies coupled to horseradish peroxidase. The reactive proteins were detected using Pierce ECL Substrate (ThermoScientific) followed by exposure to hyperfilm MP (Fisher Scientist).
